# An analysis of actions to promote health in underprivileged urban areas: a case in Brazil

**DOI:** 10.1186/1471-2296-14-80

**Published:** 2013-06-13

**Authors:** Érika Cardoso dos Reis Moreira, Gisele O’Dwyer

**Affiliations:** 1 National School of Public Health / Oswaldo Cruz Foundation (Escola Nacional de Saúde Pública/Fundação Oswaldo Cruz), 1480, Leopoldo Bulhões Avenue, Rio de Janeiro, 21041-210, Brazil

**Keywords:** Primary health care, Family health strategy, Health promotion, Health policy

## Abstract

**Background:**

Two policies stood out in the 2000s geared towards changing the care model adopted in Brazil: The National Policy on Primary Health Care, based on a family health care model, and the National Policy on Health Promotion.

The aim of this study was to analyze health promotion actions developed by family health care teams in the municipality of Belford Roxo. This town was chosen by virtue of its “below average” level of primary health care services offered in relation to other municipalities in Rio de Janeiro state.

**Methods:**

The following methodological strategies were employed: analysis of health systems, document analysis (2010 Annual Health Schedule and 2010 Annual Management Report), participant observation and interviews with nine health care professionals in the region of study, namely: the manager of the Regional Health Polyclinic (responsible for health care actions in the region), and nurses belonging to the eight family health teams. Giddens’ Theory of Structuration was used for analysis of the results.

**Results:**

Varying levels of health care activity were found, indicating that the managers have been either unable or lacked the commitment to perform the proposed actions. From a structural point of view, 87.5% of the teams were incomplete. Also of particular note was the lack of any physicians in the teams, which, despite its detrimental effect, was regarded by the interviewees as “natural”.

Strong political party influence in the area hindered relations between the team and the local population. Health education, especially through lectures was the main health promotion activity picked up in this study.

No cross-sectorial or public participation actions were identified. Connections between the teams for sharing responsibilities were found to be very weak.

**Conclusion:**

In addition to political factors, there are also structural limitations such as a lack of human resources that overburdens the teams’ daily activities. From this point of view, the political context and lack of professionals were restrictive factors for health promotion.

Belford Roxo is not necessarily representative of other experiences in Brazil. However, problems such as patronage, political manipulation, poverty and incipient cross-sectorial actions are common to other Brazilian towns and cities.

## Background

Historically speaking, health care has been focused on the formulation and implementation of policies that, over the course of time, have produced positive results for health.

Brazil made a major political investment toward qualifying its health care when it proposed the Unified Health System (SUS), created and enacted in the 1988 Brazilian Constitution as a citizen’s right and duty of the State. This health care proposal was grounded on the principles of it being public, universal, comprehensive, equal, decentralized and involving social control and participation.

Over the course of more than 20 years, the SUS has been implemented with serious financial and managerial difficulties, but has also led to unquestionable improvements of health indices and access to health services [1].

Two main policies aimed at changing the care model adopted in Brazil can be identified: The National Policy on Primary Health Care (PNAB), based on a family health care model, and the National Policy on Health Promotion (PNPS).

Primary Health Care is regulated by a national policy, which defines it as “a set of individual and collective health care actions, encompassing health protection and promotion, disease prevention, diagnosis, treatment, rehabilitation, damage control and health maintenance with the objective of developing comprehensive care that impacts on people’s health and autonomy and on the determinant factors and conditions for collective health.” [2], p. 3. This policy established Primary Health Care as the main gateway to the health service system and reasserts its importance in the SUS care framework.

Under the PNAB, the Family Health Strategy (ESF) is the priority and core model for reorganizing and ordering all SUS health care networks. The policy is built on the system’s principles and guidelines and aims to contribute toward effective universality, comprehensiveness, equality, access, coordinated care, trust and humanization [2]. The Health Promotion Policy was heavily influenced by the Ottawa Charter (1986), which defined that health is constructed through caring for oneself and for others, the ability to make decisions and have control over one’s life circumstances, and through the struggle to ensure that society provides the conditions that enable everyone to achieve health [3]. The Ottawa Charter, inspired by the principles of the Alma-Ata Declaration (1978) and by the goal of “Health for all by the year 2000”, resulted from the First International Conference on Health Promotion, and officially stated the finding that the main determinants of health lay outside the treatment system. This document proposed the notion of health as quality of life, resulting from a complex process conditioned by various factors, such as diet, social justice, the ecosystem, income and education [4].

In Brazil, Health Promotion was proposed as a distinguishing element of the new health care model, spearheaded by the Family Health Strategy.

It is important to highlight that the PNAB and the PNPS were published in the same period (March 2006), in the same historical and political context. The Ministry of Health published the National Policy on Health Promotion [5] with the intention of implementing the agenda which it had been promoting for some time, materializing the political will to reorganize health care practices. Health promotion, therefore, proposes changes in at least three spheres:

•In health care, where services are demedicalized and redirected in such a way as to receive and help individuals and groups build autonomy;

•In local development and management, where cross-sectorial actions for health and quality of life are implemented, as a result of, and motivation for the empowerment of population groups and individuals;

•In the national development policies and model, where the determinant factors for equality are actually defined [6].

Health Promotion is, therefore, presented as the opportunity not to formulate an agenda for the improvement of the SUS, but also to point toward a new agenda, redefining health policy for the new millennium and resurrecting the flag for public health reform to promote change and fight social inequalities; actions required to ensure health and dignified life for the people [6].

### The reorientation of primary care in Brazil

The debate on primary health care took on greater international magnitude in the 1970s. Questions arose which challenged the interventionist and specialist hegemonic medical model, with over-fragmented care and little impact on improving public health conditions [7].

At the 1977 World Health Assembly, the World Health Organization (WHO) proposed “Health for All by the Year 2000” as the main target for governments and the institution itself. In 1978 the 1^st^ International Conference on Primary Health Care was organized by the WHO in partnership with UNICEF (United Nations Children’s Fund) in Alma-Ata, Kazakhstan. At this conference primary health care was adopted as a strategy to achieve the Health for All by the Year 2000 goal and identified as a fundamental component of any effective health care system. Whereas the notion of primary care defended by the WHO and promoted in the wake of Alma-Ata pointed to comprehensive care, critics of that concept, interpreted as idealistic, triggered the promotion of a selective proposal, which won the support of other international agencies, including UNICEF itself, which had supported the Alma-Ata Conference [8].

Therefore, the Alma-Ata Conference is considered a milestone in the broad discussion on Primary Health Care (PHC), and the Bellagio Conference, held by the Rockefeller Foundation in Italy in 1979, with the theme “*Health and Population in Development”*, can be seen as another milestone, as that event marked the emergence of tension between these two interpretations of PHC. The notion of selective primary care introduced a new perspective, referring to a package of low-cost technical interventions to tackle the main diseases that afflicted the populations of developing nations [8].

The idea of selectivity was promoted by the World Bank (WB), which defended the concept of a “basket” of health services. It suggested that the public sector should only be responsible for providing a minimum set of essential actions to those who cannot afford health care costs [9].

In Canada there was the shift to outpatient care in the 1980s and early 1990s, with neoliberalism in full swing. Primary health care in that country was a strategy to rationalize services, reduce costs and facilitate care access and coordination, in line with the World Bank and its minimum service package [10].

European Union countries in the 1990s also witnessed the reorganization of primary care services being driven to reduce health spending and meet the demands of a changing epidemiological profile, as well as to promote coordinated service provision through various care levels [11].

The Brazilian SUS was created in 1988, in revolutionary fashion and despite the economic and political context. Due to the international diffusion of the notion of selective primary health care in the 1980s, the term Basic Health Care was coined in Brazil to distinguish the country’s proposal for universal and comprehensive care from the selective concept.

In 1991, the Ministry of Health regulated the Community Health Agents Program (PACS) based on trials developed mainly in the Northeast of the country. The Community Health Agents (ACS) are residents of the service coverage areas, trained to work with 100 to 250 families, totaling no more than 750 people. Their work is supervised and assessed by a nurse. There are no physicians in the PACS teams. Initially, very little progress was made in terms of population coverage of the program. Expansion occurred mainly in small municipalities in the North and Northeast regions, in areas where public primary care networks were practically non-existent [7]. The aim was to close care gaps, initially introducing these professionals as the Family Health Program (PSF) was being set up. The Family Health Program was singled out as a priority policy in the 1990s, and pushed harder from 1995 onwards.

The prioritization of the PSF was reflected by the adoption of a specific financial incentive [12] aimed to change traditional care patterns on a continental scale, strengthening primary health care actions in the SUS, proposed as a transformative practice.

With this incentive provided by the Ministry of Health, the Family Health Program grew across Brazil, with 3,062 family health teams implanted in 1,134 municipalities in 1998. Despite the early inclusion of towns on the outskirts of metropolitan regions, from 2000 onwards the program expanded in smaller municipalities, which situation has begun to change recently [13,14]. In 2004, the northeastern, mid-western and southern regions of Brazil had the highest proportions of PSF coverage, approximately 55%, 41% and 38% of the population, respectively, followed with the northern and southeastern regions, with 34% and 30%. From 1998 to 2004 there was a significant growth in all regions, but with greater coverage in municipalities with a low Human Development Index (HDI). In 2011, the number of family health teams reached 31,981 in 5,279 municipalities [13].

Despite the growth over the past few years, there will probably always be some discrepancy between the concept of Primary Health Care as designed by national policy and how it is expressed in local realities; with it being necessary to comprehend how Primary Health Care and the PSF are configured and under which conditions they are inserted and operate in local health systems [15].

Adhesion to the PSF varied at different moments of its growth, reflecting the cultural and social diversity between the different regions of Brazil. The initial growth followed a trend of coverage in poorly assisted areas, represented by small towns, beginning in North and Northeast [15-17]. Regardless of the phase of expansion of the family health strategy, the Southeast as a whole has displayed far less growth than the other regions of Brazil [15]. In Rio de Janeiro state, over the years hospitals have been prioritized for the provision of health care services, generating a unique disparity between hospitals and primary care units. In the state capital, coverage of the strategy was approximately 3% until 2008.

Because of the challenges and controversies surrounding the effectiveness of the family health strategy in metropolitan regions [15,16], for our field work we chose a municipality in the metropolitan region of the state of Rio de Janeiro. We chose the municipality of Belford Roxo, a town with a level of primary care services “significantly below” the state average [18], making it a priority municipality in relation to investment in health care, lying fourth from bottom in the state ranking. Table 1 shows the growth of ESF implementation over the years. In this table one can see that, although coverage of the Family Health Strategy is limited in the municipality, the number of teams has been increasing over time with a consequential growth in the provision of services and actions.

**Table 1:** Progress of family health strategy (ESF) coverage in the city of Belford Roxo from 1998-2011

**Year**	**Population**	**Family health teams**		
		**Implemented**	**Estimate of population covered**	**Estimate of population covered (Percentage)**
1998	408,949	0	0	0
1999	408,949	2	6,900	1.69
2000	425,194	14	48,300	11.36
2001	442,012	4	13,800	3.12
2002	442,012	6	20,700	4.68
2003	449,997	11	37,950	8.43
2004	457,201	17	58,650	12.83
2005	457,201	18	62,100	13.58
2006	480,695	23	79,350	16.51
2007	489,002	27	93,150	19.05
2008	489,002	22	75,900	15.52
2009	495,694	28	96,600	19.49
2010	501,544	29	100,050	19.95
2011	469,332	32	110,400	23.52

Source: MS/SAS/DAB and IBGE.

Despite the recent advances in the ESF, local health levels are extremely low, which could be related to structural or resource-related factors, such as the low provision of services on the network, low quality of the health services offered, and lack of human resources in health care. This municipality is extremely unequal in terms of human development. According to the FIRJAN World Development Indicator (IFDM), the municipality of Belford Roxo is one of the twelve worst out of all 92 municipalities of Rio de Janeiro state^a^.

Belford Roxo is split into 5 administrative regions, on which basis the municipal health secretariat organizes health territories, under the charge of a Regional Health Polyclinic (PRS). In each regional health division, primary health care is organized through traditional Basic Health Care Units (UBS), family health teams and a PRS.

The regional health division with the most structural resources was studied, with it being understood that those resources would provide the basis for more structured work. The region studied had one Regional Health Polyclinic (the first in the municipality), one Family Health Support Unit (NASF)^b^, 8 Family Health Teams, oral health teams, 5 Basic Health Care Units, 1 Referral Center and 1 Municipal Hospital. This region has recently received investments to tackle these problems.

In addition to the analysis of official data, the study also involved reviews of the 2010 Annual Health Schedule (PAS) and Annual Management Report (RAG). The examination of these documents intended to relate managerial guidelines for health promotion practices to the field work. We prioritized questions that referred to activities carried out by health teams both individually and collectively and also questions that described the integration between teams and with the Regional Health Polyclinic of Heliópolis.

In this paper we will review how primary health care, a policy that remained a Ministry of Health priority throughout the 2000s, incorporated this other priority directive: health promotion. We analyzed health promotion actions developed by family health care teams in the municipality of Belford Roxo.

## Methods

The following methodological strategies were employed: analysis of health systems, document analysis, participant observation and interviews with nine health care professionals in the study region, namely: the manager of the Regional Health Polyclinic (responsible for health care actions in the region), and nurses belonging to the eight family health teams. We began the field work by observing group meetings and health education activities over the course of a month, according to the eight family health teams’ schedule. Forty hours of participant observation were distributed among the teams. These participant observation activities led the conclusion that the nurses were key to the study, as well as the director of the Regional Health Polyclinic.

Nurses were selected as research subjects because they represent an essential category within the family health team with the “role” of team supervisors. For Araújo (2009), nurses have acted as facilitators both in family-centered and individual-centered nursing care [19].

The PRS director was invited to be interviewed as she is responsible for planning ESF activities. All the nurses and the director promptly agreed to be interviewed. The research took place in 2011. The aim of the interviews was to investigate the notion of health promotion, which health promotion actions were performed by the teams, the integration between services and communication with other sectors, as well as the relationship between management and the ESF teams in the region.

All the interviews were recorded and transcribed. They were conducted in a room at the health care unit, with complete privacy and at a time chosen by the interviewee. The interviewees were numbered in chronological order of the interviews. The research group analyzed the content of the interviews through repeated readings of the transcriptions, listening to the recordings and reviewing the notes. Comparing codes with discursive excerpts and quotes can guarantee the reliability and validity of the interview codification process.

Giddens’ Theory of Structuration was used for analysis of the results [20]. According to Giddens, social practices can be seen as procedures, methods or skilled techniques appropriately executed by social agents using rules and resources. Therefore, agents are largely free to take action, but are always conditioned by the structural resources available. Giddens sees social practices as structured within this duality of social object and individual action, and rejects the dominance of either extreme.

Actors use their knowledgeability to create routine practices or produce changes depending on the ‘circumstances of the action’. These are the ways in which social phenomena and materials facilitate or restrict human action, illustrating the duality of the structure [20].

The structure is formed by rules and resources. The normative aspect of the rules corresponds to practices from the perspective of rights and duties and the ways in which the practices can be executed. The semantic dimension of the rules corresponds to the qualitative and procedural meaning of the practices, associated to their performance. The resources are the facilities or power bases to which the agent has access and that he manipulates to influence interaction with others. These resources may be authoritative (position or office held, for example) or allocative (material) [21].

Giddens believes that agents’ actions are contextualized in time and space and depend on the availability of rules and resources. Agents are always competent at engaging these resources and the process of structuration occurs as the means and the result of the agent’s action and the engagement of the resources [20].

Access to the agent may come about through discursive consciousness or practical consciousness. The interviews were aimed at giving expression to the discursive consciousness of the actors. Access to the practical consciousness was obtained through the participant observation, addressing the agent’s perceptions, culture and professional practice.

The theory of structuration is extremely useful to demonstrate structural changes over a longer time span; however it can also show how players engage resources in microsocial spaces, whether intentionally or not.

This study was submitted for review by the ENSP Ethics Committee and was duly approved; case number 0236.0.031.000-10. The subjects of this research were informed of its objectives, benefits and risks, and were offered the choice to participate or not. An informed voluntary consent form was provided to all the interviewees; information was provided under terms of strict confidentiality and anonymity.

## Results

The Health Care Plan, Annual Health Care Schedule and Annual Management Report were the focus of the document analysis. The organization of the Annual Health Schedule is represented in Table 2.

**Table 2:** Annual health planning organization, 2010 – Belford Roxo/RJ, 2011

**Benchmarks**	**Guidelines**
Benchmark I: determinants of health and health conditioning factors	Guideline 1: Expanding people’s access to healthcare services and improving the quality of healthcare actions
	Guideline 2 – Organizing and training the Superintendent’s for Basic Healthcare Office Staff
	Guideline 3: Guaranteeing comprehensive care by performing actions in the life cycle prioritized by Life Pact goals
	Guideline 4: Guaranteeing that people’s healthcare needs will be met through technical, interdisciplinary, strategic and scheduling actions
	Guideline 5: Improving effectiveness of specialized and reference outpatient and hospital services taking into account a Healthcare Network that is territorial, hierarchical and regional
	Guideline 6: Strengthening the pharmaceutical assistance policy
	Guideline 7: Guaranteeing the implementation of health inspection actions, preventing and controlling diseases affecting the population
Benchmark II: people’s health conditions	Guideline 1: Promoting intersectoral actions that contribute to improving life and health conditions of the population
	Guideline 1: Structuring and Training management by incorporating innovative and sustainable planning processes
	Guideline 2: Improving the process of decentralizing / regionalizing and territorializing the Healthcare network
Benchmark III: improving municipal management	Guideline 3: Organizing and regulating access to outpatient, hospital and testing services
	Guideline 4: Strengthening people’s participation and social control in SUS Management
	Guideline 5: Work management and improvements to better meet the needs of SUS users
	Guideline 6: Organizing and Strengthening health education actions
	Guideline 7: Improving the quality of Health communication and information processes
	Guideline 8: Municipal healthcare system maintenance – costs and investments

The Health Care Plan in defined in article 2 of Ministerial Directive 3.332/2006 of the Ministry of Health as an instrument to be developed based on situational analysis, presenting the planning expressed in terms of objectives, guidelines and targets for a four-year period. It should identify health policies and commitments for a specific management level, as well as forming the basis for the execution, monitoring, evaluation and management of the health system [22], p. 18. The Annual Health Care Schedule is “the instrument that supports the implementation of the intentions outlined in the Health Care Plan” and the Annual Management Report is “the instrument that presents the results achieved and offers any steering that may be necessary” [22], p.33.

In the analysis of the Annual Health Schedule, health promotion is part of the planning for some areas/sectors, such as the technical coordination of health care for the elderly, the health information, education and communication sector, worker health and technical coordination of diet and nutrition. These groups plan health promotion actions, but still in an isolated manner, with no communication neither within the health care sector nor between different sectors. Very little dialog with other sectors of local management was found and cross-sectorial relations, one of the foundations for health promotion, was disregarded in the majority of the planned actions.

When reviewing the Annual Management Report and checking the achievement of targets, including health promotion, varying levels of health care activity were found, indicating that the managers have been either unable or lacked the commitment to perform the proposed actions. The documents may function merely as an element of bureaucracy in the system We conducted the field work to investigate whether the municipality of Belford Roxo had incorporated the recommendations from a prescriptive point of view, or if that planning had influenced care workers, functioning as a rule and resource that the agents of the practice used to modify their actions [20].

## Discussion

The discourse and the local scenario of practices were analyzed from two different perspectives: in terms of the structural dimension and the organizational dimension. The structural dimension represented Giddens’ structural category, that is, the rules and resources for the action; whereas the organizational dimension represented the action of the agents based on the engagement of those resources.

### Structural dimension

In this section we will focus on the composition of the teams, their training and client allocation. Client allocation was considered a structural question, as it does not depend on the team and serves as a resource or rule to be engaged.

According to a governmental decision, a Family Health team is responsible for the health care of a population group of no more than 4,000 people who live in a defined geographical area. Family health teams consist of at least one physician, one nurse, one nursing assistant or technician, and community health agents (ACS). Each ACS should not be responsible for monitoring any more than 750 inhabitants and each team for no more than 12 ACS, operating in previously defined areas [2].

In the municipality studied, 87.5% of the teams were incomplete. One recurrent difficulty in Brazil is establishing doctors within the family health strategy, something achieved in only 12.5% of the teams studied. The SUS competes with the private health market for doctors, since most of them work under both the public and private systems. This dual commitment works to the detriment of the public sector, involving even practices of differing quality [23]. Regardless of our analysis, the lack of physicians does not seem to be the main problem with team composition; the issue is exacerbated by the fact that in many teams the prescribed working hours are not fulfilled.

It is common practice for SUS doctors to work only a fraction of their specified hours. According to Giddens’ framework, we could say that this has been a structuring factor for care actions in the SUS. This situation is illustrated by how ‘naturally’ one interviewed nurse talks about the team doctor, who works less than his contract hours but is not held accountable for it.

*“We don’t do pre-natal care because the doctor doesn’t do it… we can’t do it on our own, only if the doctor is present… and he doesn’t have any available hours.”* E2

In a study with similar objectives to this one, Beato *et al* (2011) showed how continued family health team stability over time helped improve the work process [24].

On the flip side to Beato *et al’s* study, the lack of doctors in the teams studied distorts the work of the family health strategy, as indicated by the following statement:

*“There were no doctors working at our clinic for a year and a half… We no longer have that bond we used to have, for example, in 2005, 2006, when I used to work. The doctor came every day. So we were much more integrated with the families because they had monthly appointments, one with the doctor and the next with the nurse. So we knew. I knew which children were behind with their immunizations. I had everyone in the palm of my hand … Today we’re basically following up and that’s all. So it’s not like before when we had… It was really a PSF. But not today, today the doctor no longer has the time to do house calls… She no longer has time to provide the care they should be getting.”* E8

This account reports the physical absence of the doctor and the medical rationale that the policy on primary health care and health promotion intends to overcome; that of care based purely on curative activities. Geneua *et al* (2008) discussed medical practice in primary health care and pointed out how difficult doctors found it to take an active role in the educational activities [25].

This difficulty for doctors to commit to non-care and health promotion activities was confirmed by one interviewee.

*“The doctors hardly ever participate, because they come to treat patients … it’s usually the nurses, the ACSs, the dental hygiene and the NASF staff.”* E1

In this context, Machado and Porto (2003) stated that the behavior of several actors reflects certain power structures and cultural practices that must be overcome in order to give way to other more effective ones. Even when there are resources available to facilitate the agents’ actions, the doctors fail to make use of such resources to change their practice and structure a form of care more in line with guidelines for health promotion and health care model change [26].

The population group allocated to each team varied from 400 to 1,500 families, and from 80 to 187 per ACS. It was found that 12.5% of the teams provided services to a larger population group than recommended. Bearing in mind the average family size in poor municipalities like Belford Roxo is 4.2 members, we could see that the majority of ACSs were working with less people than determined in the ministerial directive.

However, ACSs represented the main human resources shortage in the region. This was a surprising discovery, as it had not been indicated in any nationwide studies.

We found that this difference in relation to other scenarios in the country could be explained by the strong and long-standing connection between party politics and public health services in the area. No specific elements were expounded that confirmed this hypothesis; however, since the agents must live in the community, this impression remains strong. The political dispute over welfare in places like Belford Roxo is deeply rooted in the exchange of votes for personal interests. This issue has heavily interfered in the formation of trust relationships between the teams and the public.

Some units belonged to local politicians and were leased to the municipal health secretariat, and rival groups “attacked” the unit where the health team was installed as a form of aggression against the owner, their political opponent.

*“No, we don’t have any of that… Some do form a good relationship, but we have a lot of problems in this PSF…, it really does belong to a politician. The building is rented… We’re always having problems. We lose vaccines all the time because someone’s disconnected the mains over the weekend… On Friday, they broke in here… someone tried to steal things from the kitchen, stuff like that… And it makes it hard… because we’re always denying people things. We never have any vaccines (reports that disconnecting the mains is a frequent occurrence), and so we can’t provide good service, we’ve got no medications…”* E8

Local party politics has also hindered the allocation of the family health services. Some teams did not cover people who lived on the same street, but covered other more distant neighborhoods. This was a result of the allocation not taking into consideration the needs of the local population and their proximity to the unit, damaging the work process of the teams, who also take the blame for the problem. In this case, allocation, which should be a facilitating rule of ESF action, is transformed into a restrictive rule.

*......what happens here is that some streets are not listed, so this causes a huge problem for us here and for the users, who want to make appointments here and can’t. So this is a State issue when they brought the PSF here and did this, they some listed streets that are miles away… and some people who live around the corner are not listed, for example. So it’s a little complicated… people don’t understand… they want the service, they don’t understand, and they want to attack us, they think it’s our fault. So it’s a love-hate relationship, you know?”* E8

Another structuring element revealed in the research is that the health care professional most frequently present in the team, the nurse, had no prior professional experience in the family health strategy or primary care; they had either recently graduated or had previously worked in hospitals before coming to work with primary care. This is perhaps not surprising, as we are going through a phase of modification of the care model and until recently the hospital was the main setting for health care. During such a transition, staff training is required in order to build the new model.

The way in which these professionals described health promotion reflected their lack of training to work in the family health strategy.

*“… Prevention, because me offering prevention will bring about health promotion for them. I go along and do the prevention, check their pressure, give them advice.”* E7

The director of the polyclinic showed a broader understanding of the concept, seeing health promotion as incorporating education, employment, right to leisure time and acknowledging the need for cross-sectorial participation.

### Organizational dimension

In this section we will discuss cross-sectorial characteristics and the planning and performance of health promotion actions. For actions to be cross-sectorial, joint efforts are required for the planning and tackling of local issues, that is why this aspect has been categorized as an organizational dimension.

Cross-sectorial work is an essential activity for health promotion and was regarded as the use of the physical space of the social apparatus in the area. In the case of the Regional Health Polyclinic, the management created an association formed by local businessmen, families and the Catholic Church. There is a very close relationship and partnership with the Catholic Church, because the polyclinic is built on land owned and provided by the church. Apart from this partnership with the association, there are no other partnerships with structuring sectors such as education, public security, social assistance or housing. The polyclinic management reports that the partnership with the church and school consists in using collective community spaces for any talks given outside the health service.

*“Most the time they’re held here in the unit, sometimes at a church, but usually here.”* E8

Another important promotional activity is changing unhealthy living habits, such as alcohol abuse, smoking and taking drugs, unhealthy diets and lack of physical exercise. A recent study [27] investigated health promotion based on changes in lifestyle. One important finding of the study was how valuable the nursing team is to health promotion. The authors identified the patient, the health care professional and managers as actors bearing responsibilities for making lifestyle changes [27]. In our study it was evident that the responsibility-sharing bond was very weak. The nurses address questions of lifestyle changes, but always in a normative manner with very limited contextualization and planning.

Health education, especially through lectures was the main health promotion activity picked up in this study.

*“Everything we cover here is through talks, giving advice, promoting something, organize a game, have a snack so they feel welcome and talk… we use whatever is available, like leaflets … ”* E8

Here is another account of these educational activities:

*“Giving patients advice, giving the community advice on how to prevent diseases before they appear; monitoring preventions actions and not just coming to the unit when they’re ill, but rather coming beforehand to really promote health, and especially using talks, because this is how they can learn to identify anything in the early stages.”* E1

Some teams, although failing to engage in any cross-sectorial activity, do request other local sectors (such as the economic sector) to carry out activities with the public.

*“… Every time we’ve promoted any action the community has helped us; local businessmen, the church, we've held events at the church, nothing else. Whenever we want to do any social work, yes, only then, otherwise (gesticulates to say no).”* E2

Even among teams that report planning their activities, we notice that such only pertained to making appointments or scheduling activities within the health care unit, without mentioning any involvement of other sectors or actions outside that sphere.

None of the interviewees cited basing their planning or their activities on Ministry of Health documents, such as the PNPS [5], or guidelines issued by the local health secretariat or family health strategy coordinator’s office, or even on any epidemiological evidence. For the regional director, the identification of health problems depends on Ministry of Health guidance and on the physician’s ability to correctly assess patients’ conditions, acquired through practice.

We imagine that the Polyclinic should play an important role in guiding and integrating the several family health teams, action planning and team training. However, the meetings between the different teams were identified as merely a platform for the delivery of production reports.

*“We meet up when we delivery production reports. The 20*^*th*^*of every month.”* E7

As regards factors that might interfere in the performance of actions deemed to “promote health”, the nurses almost always reported lack of time, since most the teams are incomplete which leads to work overload, allied to a lack of institutional support. The lack of doctors in the teams ends up overburdening the nurses, who are charged with solving people's health problems and therefore unable to perform their own duties with the families. As well as this technical impact, there is also the symbolic importance of the doctor in the eyes of the public, who often do not have faith in the team when the doctor is not present.

*“Lack of doctors … the families don’t come if there’s no doctor… only when they need some medicine…”* E6

Mattos [28] maintains that transforming the care practices is the challenge for all the policies, with the aim of providing some response to human suffering and producing comprehensive public health care. The attitude toward suffering was represented by a degree of concern on the team’s part to meet the population’s needs.

*“If someone comes in, not an emergency, but if they’re unwell, with a high fever … we provide care for them. Sometimes we also attend to emergency cases.”* E7

The nurses observed and interviewed referred to a lack of enthusiasm and belief in the proposed reorientation of the model. However, they were aware of how important their work was. They acknowledged the heavier burden imposed by the doctors’ absence, but did not criticize such absence, as if it were a structuring rule of the ESF, not being able to count on doctors’ participation.

*“I don’t think I’ve helped very much… I couldn’t provide particularly good care because we didn’t have a doctor, so it’s all about the consultations … and when you’re alone you can't get everything across… it’s good when there are the two of you speaking to the public, the doctor and the nurse together, then it’s completely different.”* E6

However, there are also cases where the unit has no bond with the population and contact occurs mainly to meet health care needs at a given moment in time. It is easy to perceive how this habit of seeking care only when really necessary weakens the bond between the team and user, as well as the health promotion outlook, especially when there is no doctor figure participating in the team activities. This practice is perpetuated because the doctor’s work in the team is usually limited to exclusively curative care.

## Conclusions

We opted to study a regional health care division with the largest care network in a very poor municipality, with major historical health care deficiencies. The result of the study cannot be considered representative of the municipality, which is still implementing the Family Health Strategy framework. However, the aim was to demonstrate the complexity in modifying a health care model in a poor metropolitan municipality, like many others in Brazil, despite the structural investment in the ESF.

Primary health care is acknowledged as an innovation of global significance, which Brazil has adopted as its model for the SUS. Structuring a change in the care model is a dynamic process that takes places over the course of time as the actors involved engage the structural resources. It is our understanding that the model based on primary care and health promotion is a form of structuring a new health care proposal; hence our choice to analyze the actors’ actions in relation to this new model, even in situations of co-presence and over a short period of time.

Through documental analysis of the Annual Health Schedule and Annual Management Report for 2010, we could identify that the health teams and professionals were encountering difficulties in incorporating many of the proposed actions.

In general, the objectives and targets proposed in the plans were not met due to a lack of technical cooperation between those responsible for executing the actions, an unequal distribution of resources and a lack of political coordination and partnerships to complete the actions. Another challenge is to implement continuous assessment of actions with the presence of the health teams or team representatives, since activity reports and documents are currently prepared by the technical coordination without any involvement of the local actors responsible for the actions. Therefore this document is a management resource to support the action of those professionals which has not been employed to its full ‘facilitating’ potential.

The health care teams’ promotional actions are often unplanned, as training deficiencies limit and steer their actions toward treating disease.

When health promotion actions are performed, the involvement of other social actors is still limited. This study found out that other sectors, such as education, the environment, labor and social assistance, are not integrated into activities aimed at improve people’s health. Additionally, partnerships occur on the basis of availability of venues for teams to perform their desired activities. In other words, there is no cross-sectorial support network focused on public health promotion. As a result of this lack of a support network allied to an unfavorable context such actions become increasingly less frequent in the teams’ daily routines. Despite these criticisms of the lack of cross-sectorial actions, we understand that this is a role that should also be shared with the NASF and other structures within the municipality.

The study revealed the presence of political factors hindering the establishment of partnerships with other sectors and even the smooth running of the unit. When a health care facility cannot offer the public basic actions like medical care and immunization, its task to develop partnerships and trust-based relationships becomes significantly harder. This situation has prevented the health teams operating in this context from becoming a reference for the local population. These teams have therefore been unable to reinforce any community action as subjects in the self-care process.

Another particular feature of this region, which clearly illustrates how the microsocial relations are structuring, was the way in which the client allocation was allowed to be executed. The exclusion of neighboring streets from the health units is a new, locally-established rule that has generated dissatisfaction and delegitimized the potential bond intended by the ESF.

In addition to political factors, there are also structural limitations such as a lack of human resources that overburdens the teams’ daily activities. Lack of physicians or reduced hours worked by those professionals is a limiting factor for the execution of health promotion activities, however the nurses refrain from criticizing this role, restricted to curative practice, which hinders the formation of a bond between the public and the team. From this point of view, the political context and lack of professionals were restrictive factors for health promotion.

Therefore, as regards health promotion actions in primary care in Belford Roxo, the results of this study indicate that tackling issues as structural as poverty and party political coercion requires a better qualified professional, integrated into his work and supported by other instances, such as polyclinics and health departments. There are several regions in Brazil with serious socio-environmental weaknesses and governmental proposals have addressed the problem from a normative perspective. However, these resources are unable to change the care model and promote health in situations of such difficulty, as reported in the case of this study.

Despite the difficulties identified in nurses’ practice, these professionals are crucial to the success of the ESF and health promotion.

A limitation of this study is the acknowledgment that the municipality of Belford Roxo does not necessarily provide a fair representation of the experiences throughout Brazil, given the heterogeneous nature of the country and of the implementation of the policy in local contexts. However, problems such as patronage, political manipulation, poverty and incipient cross-sectorial actions are common to other Brazilian towns and cities. Another limiting factor was the choice to interview the nurses, but not the rest of the team members to understand the teamwork proposed for primary care.

We recommend the assessment of other regions with similar characteristics, as well as regions in different social and care scenarios. It is our understanding that further qualitative studies are necessary in order to understand how the ESF is being structured as an organizational basis of the care model.

Other experiences in Brazil have reported positive results, based on heavy investment in partnerships, training and health care funding [29].

We hope that the matters addressed here will motivate a fresh debate on the topic of health promotion and, above all, the various ways in which better living and health conditions can be achieved for individuals.

## Endnotes

^a^IFDM is an annual study conducted by the FIRJAN System (Rio de Janeiro State Federation of Industries) and that follows the development of all 5,564 Brazilian municipalities in three areas: Employment, Income, Health and Education. It is based exclusively on official public statistics, provided by the Ministries of Labor, Education and Health.

^b^The Family Health Support Unit (NASF) consists of teams that are composed of professionals from different areas. They provide support and work in partnership with Family Health professionals, focusing on health practices in areas under the responsibility of the FH team NASF is an innovative strategy that aims to help, expand and improve health care and management in Basic Care/Family Health.

## Abbreviations

ACS: Community health agent; APS: Primary health care; WB: World bank; ESF: Family health strategy; HDI: Human development index; IFDM: FIRJAN World development indicator; FIRJAN: Rio de Janeiro State Federation of Industries; NASF: Family health support unit; WHO: World health organization; PAS: Annual health schedule; PNAB: National policy on basic health care; PNPS: National policy on health promotion; PSF: Family health program; RAG: Annual management report; SUS: Unified health system (Sistema Único de Saúde); UNICEF: United nations children’s fund.

## Competing interests

The authors declare to have no conflict of interests.

## Authors’ contributions

ECRM made a substantial contribution to the original idea, design and data collection, analysis and interpretation, and wrote the manuscript. GO made a substantial contribution to the original idea, design, and data analysis and interpretation, and wrote the manuscript. All the authors have read and approved the final manuscript.

## Authors’ information

GO is a researcher belonging to the research group that investigates the “Formulation and implementation of public policies and management of health systems – theoretical and methodological approaches in public policy analysis”. ECRM is a Master’s degree student in Public Health at the National School of Public Health / Oswaldo Cruz Foundation.

## Pre-publication history

The pre-publication history for this paper can be accessed here:

http://www.biomedcentral.com/1471-2296/14/80/prepub
